# A Global Bibliometric Analysis of the Top 100 Most Cited Articles on Early Thoracotomy and Decortication in Pleural Empyema

**DOI:** 10.7759/cureus.72800

**Published:** 2024-10-31

**Authors:** Vishal V Bhende, Amit Chaudhary, Soumya Madhusudan, Viral B Patel, Mathangi Krishnakumar, Amit Kumar, Shradha U Patel, Swati Roy, Bhargav A Gandhi, Saptak P Mankad, Ashwin S Sharma, Jaimin P Trasadiya, Mamta R Patel

**Affiliations:** 1 Pediatric Cardiac Surgery, Bhanubhai and Madhuben Patel Cardiac Centre, Shree Krishna Hospital, Bhaikaka University, Karamsad, IND; 2 Vascular Surgery, King George's Medical University, Lucknow, IND; 3 Anaesthesiology, St John’s Medical College Hospital, Bengaluru, IND; 4 Radiodiagnosis & Imaging, Pramukhswami Medical College & Shree Krishna Hospital, Bhaikaka University, Karamsad, IND; 5 Anaesthesiology, St. John’s Medical College Hospital, Bengaluru, IND; 6 Pediatric Cardiac Intensive Care/Pediatric Intensive Care Unit (PICU), Bhanubhai and Madhuben Patel Cardiac Centre, Shree Krishna Hospital, Bhaikaka University, Karamsad, IND; 7 Pediatrics, Pramukhswami Medical College & Shree Krishna Hospital, Bhaikaka University, Karamsad, IND; 8 Epidemiology and Public Health, Amrita Patel Centre for Public Health, Bhaikaka University, Karamsad, IND; 9 Internal Medicine, Dev Medical Hospital, Vadodara, IND; 10 Internal Medicine, Gujarat Cancer Society Medical College, Hospital and Research Centre, Ahmedabad, IND; 11 Central Research Services, Bhaikaka University, Karamsad, IND

**Keywords:** decortication, open thoracotomy, pleural empyema, pleural infection, video-assisted thoracoscopic surgical decortication

## Abstract

Most pleural empyema cases are linked to pneumonia, a substantial fraction of patients present with empyema without any association to pneumonia. The occurrence of empyema caused by tuberculosis (TB) is increasing in regions where TB is prevalent.

In May 2024, a bibliometric analysis was conducted involving the screening of 7,620 articles sourced from Google Scholar. Google Scholar was selected for its comprehensive nature, encompassing articles indexed in prominent databases like Web of Science, Scopus, and PubMed. This allowed access to significant studies that might be overlooked if they were not indexed by these databases. Articles were selected based on their citation count and specific inclusion criteria, focusing on early thoracotomy and decortication in pleural empyema. Two authors (VB and MK) independently conducted a thorough screening and data collection.

The hundred top articles published from 1945 to 2015, garnered a total of 16,928 citations. These articles were written by 93 distinct first authors from 22 countries and 83 institutions, and were featured in 35 journals. The primary categories of literature included those describing the disease characteristics, features, causes, and types of pleural empyema, as well as various treatment modalities and management strategies, each constituting 37% of the literature. Additionally, pediatric empyema was a focus in 11% of the articles. The present analysis highlights publication trends, identifies gaps in the literature, and suggests areas for future research, serving as a valuable resource for guiding upcoming studies on early thoracotomy and decortication in pleural empyema.

## Introduction and background

The treatment of thoracic empyema has significantly progressed since it was first addressed by Hippocrates [1,2]. Empyema is known to be associated with considerable morbidity and, in the worst cases, causes significant mortality [3]. Empyema progresses from exudative to fibrinopurulent, and finally to the organizing stage. Successful treatment during the early stages typically includes antibiotics and closed chest drainage. Up to 80% of small uniloculated empyema may be successfully treated with antibiotics alone [4]. However, antibiotic efficacy declines in the fibrinopurulent and organizing stages as fluid locules and pleural peel develop [2,4].

The initial failure of non-invasive and minimally invasive treatments, such as video-assisted thoracoscopic surgery (VATS), requires surgical intervention in the form of open thoracotomy and decortication [5]. Surgery has shown effectiveness in multi-loculated empyema after being carefully weighed against its associated risks. However, empyema treatment outcomes are still being debated and determined primarily by the surgeon's experience [5,6]. The stages of pleural empyema reflect a continuous spectrum of events rather than separate phases [4]. Some patients may have sufficient time to consider surgical options if medical therapy fails, while others may require immediate surgery due to rapid disease progression and health deterioration. Therefore, the initial therapeutic decision for empyema should be made with careful consideration.

It is widely accepted to opt for thoracoscopic treatment in the fibrinopurulent stage of empyema, but the treatment of chronic cases remains contentious. Studies have reported variable findings of very low to high morbidity and mortality by surgical interventions in chronic cases of empyema [5,6,7]. Some studies highlight the importance of minimally invasive techniques (MIT) in chronic empyema [5].

Bibliometric analysis is a well-established research method to assess the impact of published research in a specific subject area to identify trends in scholarly output. A bibliometric analysis for the importance of early thoracotomy and decortication in pleural empyema has not been reported previously. The present study aims to provide an extensive review of the 100 most influential and top-cited publications to highlight the publication trends, key research areas, and identification of gaps in existing literature. This approach will shed light on leading research to inform future global multicenter research in this field.

## Review

Methodology

In May 2024, the Google Scholar database was searched for relevant articles on the topic. The search was conducted using specific terms, including "surgical decortication," "decortication," "open thoracotomy," "video-assisted thoracoscopic surgery," "pleural empyema," "pleural infection," and "VATS."

The initial search yielded 7,620 articles. These articles were sorted by citation count (CC) in descending order. Only articles primarily discussing early thoracotomy and decortication were included in the analysis, while those outside this scope or with inaccessible full texts were excluded.

A thorough selection process was conducted, with two independent authors (VB and MK) reviewing the titles, abstracts, and full texts of the search results to identify relevant papers. The process entailed a thorough review of 357 articles that met the inclusion criteria, from which the top 100 were chosen. Relevant data were then extracted from these selected articles, and any discrepancies during the selection and extraction phase were resolved through consensus. The extracted data included the article title, publication year, journal title, CC, first author's name, and country. The categorical distribution of the articles was done based on treatment methods, disease characteristics, diagnosis, staging, and prognosis, along with clinical trials and review articles, including research on pediatric empyema. Treatment options like VATS, fibrinolytic therapy, tube thoracostomy, decortication, and open thoracotomy were seen in the literature. Disease presentations associated with these interventions, including parapneumonic effusions, empyema thoracis, post-traumatic empyema, fibrinopurulent empyema, infectious empyema, and post-pneumatic pleural empyema, were incorporated. Prognostic indicators such as radiological staging, severity scoring, disease classification, imaging techniques, sonographic predictors, risk assessments, predictive and prognostic factors, and pediatric-specific considerations in empyema management were also included. Additionally, the bibliometric analysis integrated clinical trials and review articles referencing these topics to assess trends and identify research gaps. The 100 most cited articles on early thoracotomy and decortication in pleural empyema were evaluated by bibliometric analysis.

Results

The top 100 most cited articles had a total of 16,928 citations. Each article had an average of 169.28 citations, with a range of 82 to 650 citations. Selected articles were published between 1945 to 2015 with 93 authors and taken from 35 separate journals. Contributors of the articles were from 22 countries and 83 institutions (Table 1).

**Table 1:** Top 100 most cited articles on early thoracotomy and decortication for management of pleural empyema based on the number of citations in decreasing order # : serial number, PY: publication year, CC: citation count, CPY: citation per year, USA: United States of America, UK: United Kingdom Journal title A: Proc Am Thorac Soc, B: CHEST, C: Eur Respir J, D: Am J Respir Crit Care Med, E: J Pediatr Surg, F: Am Rev Respir Dis, G: Eur J Cardiothorac Surg, H: Pediatrics, I: Clin Infect Dis, J: J Thorac Cardiovasc Surg, K: Emerg Med J, L: Radiology, M: Ann Thorac Surg, N: J Thorac Oncol, O: Interdiscip Cardiovasc Thorac Surg, P: Am J Surg, Q: Cochrane Database Syst Rev, R: Pediatr Pulmonol, S: Pediatr Infect Dis J, T: Arch Dis Child, U: Pediatr Radiol, V: Thorax, W: Surg Endosc, X: J Ultrasound Med, Y: Int J Clin Pract, Z: ANZ J Surg, AA: Injury, AB: Respiration, AC: J Trauma Acute Care Surg, AD1: PLoS ONE, AE: Dis Chest, AF: Archives of Surgery, AG: Clin Med Insights Circ Respir Pulm Med,  AH: Ann Surg, AI: Pediatr Surg Int Category 1: parapneumonic effusions, 2: empyema, 3: review, 4: treatment, 5: pediatric empyema, 6: video-assisted surgery, 7: editorial, 8: hemothorax, 9: clinical trial, 10: fibrinolytic therapy, 11: adult, 12: retrospective chart review, 13: thoracostomy, 14: trauma, 15: chest drainage, 16: infections, 17: thoracoscopy, 18: debridement, 19: oncology, 20: decortication, 21: consensus statement, 22: thoracotomy, 23: diagnosis, staging, and prognosis, 24: surgery, 25: pneumonia, 26: case series, 27: antibiotics, 28: pleural diseases, 29: cost-effectiveness analysis

#	PY	Authors	Journal	Article Title	Category	Country	Citation Count (CC)	Citation Per Year (CPY)
1	2006	Light	A	Parapneumonic Effusions and Empyema [8]	1, 2, 3	USA	650	36.11
2	1997	Wait et al.	B	A Randomized Trial of Empyema Therapy [9]	1, 4	USA	498	18.44
3	1997	Hamm et al.	C	Parapneumonic effusion and empyema [10]	1, 2, 3	Germany	425	15.74
4	2006	Sonnappa et al.	D	Urokinase and VATS in Childhood Empyema [11]	4, 5, 6	UK	399	21.17
5	1995	Light	B	A New Classification of Parapneumonic Effusions and Empyema [12]	1, 2, 7	USA	372	12.83
6	1996	Landreneau et al.	B	Thoracoscopy for Empyema and Hemothorax [13]	2, 6, 8	USA	352	12.57
7	2009	St. Peter et al.	E	VATS versus fibrinolysis for pediatric empyema [14]	5, 6, 9, 10	USA	323	21.53
8	1993	Sahn	F	Management of Complicated Parapneumonic Effusions [15]	1, 2, 3, 4	USA	319	10.29
9	2007	Molnar	G	Surgical treatment of adult empyema [16]	3, 11	Hungary	295	17.35
10	2005	Luh et al.	B	VATS in Complicated Parapneumonic Effusions or Empyemas [17]	1, 6, 12	Taiwan	291	15.32
11	2005	Avansino et al.	H	Primary Operative Versus Nonoperative Therapy for Pediatric Empyema: A Meta-analysis [18]	3, 5	USA	287	15.12
12	2007	Sahn	I	Diagnosis and Management of Parapneumonic Effusions and Empyema [19]	1, 2, 3	USA	260	15.29
13	1985	Lemmer et al.	J	Modern management of adult thoracic empyema [20]	2, 4, 11	USA	252	6.46
14	1995	LeMense et al.	B	Empyema Thoracis Management [21]	2, 4, 12	USA	245	8.45
15	1999	Cassina et al.	J	VAT in stage-based pleural empyema management [22]	2, 4, 6	Switzerland	242	9.68
16	2000	Bailey	K	Tube thoracostomy complications in trauma [23]	2, 4, 13, 14	USA	236	9.83
17	1988	Silverman et al.	L	Thoracic empyema: management with image-guided catheter drainage [24]	2, 4, 15	USA	233	6.47
18	1991	Ashbaugh	B	Morbidity and Mortality in Empyema Thoracis [25]	2, 4	USA	233	7.06
19	2001	Waller et al.	M	Thoracoscopic decortication: VAS in chronic postpneumonic pleural empyema [26]	2, 6	UK	219	9.52
20	2000	Chen et al.	B	Bacteriology of Acute Thoracic Empyema [27]	2, 16	Taiwan	217	9.04
21	1993	Kern et al.	E	Thoracoscopy in pediatric empyema [28]	2, 4, 5, 17	USA	214	6.9
22	1994	Robinson et al.	M	Fibrinolytic treatment of empyemas [29]	2, 10	USA	209	6.97
23	1991	Ridley et al.	M	Thoracoscopic management of empyema [30]	2, 4, 18	UK	207	6.27
24	1998	Striffeler et al.	M	VATS for Fibrinopurulent Pleural Empyema [31]	2, 6, 18	Switzerland	199	7.65
25	1994	Pothula et al.	B	Early Aggressive Surgical Management of Parapneumonic Empyemas [32]	1, 4	USA	189	6.3
26	2012	Lang-Lazdunski et al.	N	Management of Patients with Malignant Pleural Mesothelioma [33]	4, 19, 20	UK	186	15.5
27	1996	Temes et al.	B	Intrapleural Fibrinolytics in Empyema Management [34]	2, 4, 10	USA	183	6.54
28	2015	Scarci et al.	G	EACTS expert consensus statement for surgical management of pleural empyema [35]	4, 6, 16, 21	UK	182	20.22
29	2010	Chambers et al.	O	VATS decortication vs. open surgery in adult empyema management [36]	1, 2, 6	UK	174	12.43
30	2006	Kurt et al.	H	VATS Versus Conventional Thoracostomy Drainage in Pediatric Parapneumonic Effusions [37]	1, 4, 5, 6	USA	172	9.56
31	1996	Bryant et al.	I	Pleural Empyema [38]	2, 3	USA	168	6
32	1989	Eddy et al.	P	Empyema thoracis in patients undergoing emergent closed tube thoracostomy for thoracic trauma [39]	2, 13, 14	USA	166	4.74
33	2007	Chan et al.	M	Empyema: VATS versus Thoracotomy [40]	6	China	166	9.76
34	2005	Coote et al.	Q	Surgical versus non‐surgical management of pleural empyema [41]	2, 3, 4	UK	165	8.68
35	1976	Kish et al.	M	Early Thoracotomy for Chest Trauma [42]	14, 22	USA	163	3.4
36	2010	Tong et al.	M	Outcomes of VATS Decortication [43]	2, 6, 20	USA	157	11.21
37	2005	Lardinois et al.	M	Predictive Factors for Conversion Thoracotomy in Empyema [44]	6, 16, 23	Switzerland	155	8.16
38	2005	Misthos et al.	G	Early use of intrapleural fibrinolytics in the management of postpneumonic empyema [45]	1, 2, 10	Greece	148	7.79
39	1999	Huang et al.	B	Predicting Factors for Outcome of Tube Thoracostomy in Complicated Parapneumonic Effusion or Empyema [46]	1, 13, 23	Taiwan	147	5.88
40	2005	Jaffe et al.	R	Management of empyema in children [47]	4, 5	UK	147	7.74
41	2006	Wurnig et al.	M	VATS for Pleural Empyema [48]	2, 6	Austria	146	8.11
42	1984	Coselli et al.	P	Reevaluation of early evacuation of clotted hemothorax [49]	4, 8, 14	USA	145	3.63
43	1991	Smith et al.	M	Empyema thoracis [50]	2, 4, 16	Australia	145	4.39
44	1991	Hoff et al.	S	Parapneumonic empyema in children [51]	1, 5, 16, 23	USA	141	4.27
45	2003	Cohen et al.	J	Primary thoracoscopic treatment of empyema in children [52]	5, 17	UK	141	6.71
46	1997	Lawrence et al.	M	Thoracoscopic Debridement of Empyema Thoracis [53]	2, 6	UK	138	5.11
47	2003	Baranwal et al.	T	Empyema thoracis: a 10-year comparative review of hospitalised children from South Asia [54]	5, 16, 20, 22, 24	Nepal	138	6.57
48	2004	Gates et al.	E	Drainage vs. fibrinolytics vs. surgery in pediatric empyema [55]	4, 5	USA	138	6.9
49	2009	Calder et al.	U	Imaging of parapneumonic pleural effusions and empyema in children [56]	2, 3, 5, 23	UK	138	9.2
50	1992	Storm et al.	V	Treatment of pleural empyema secondary to pneumonia [57]	2, 4, 25	Denmark	137	4.28
51	2007	Solaini et al.	W	VATS in pleural empyema [58]	2, 6	Italy	137	8.06
52	1997	Chan et al.	E	Empyema thoracis in children [59]	2, 4, 5	Canada	136	5.04
53	2000	Chen et al.	X	Septation and acute thoracic empyema [60]	2, 23	Taiwan	136	5.67
54	2001	Tuncozgur et al.	Y	Intrapleural Urokinase in the Management of Parapneumonic Empyema: A Randomised Controlled Trial [61]	1, 9	Turkey	136	5.91
55	2006	Bilgin et al.	Z	Early Aggressive Management of Empyema Thoracis [62]	1, 6	Turkey	136	7.56
56	2008	Eren et al.	AA	Posttraumatic empyema [63]	4, 14, 23	Turkey	135	8.44
57	2008	Koegelenberg et al.	AB	Parapneumonic Pleural Effusion and Empyema [64]	1, 3	South Africa	135	8.44
58	1997	Mandal et al.	AC	Posttraumatic Empyema Thoracis [65]	4, 14, 16	USA	134	4.96
59	2004	Gates et al.	E	VATS for pediatric empyema [66]	3, 5	USA	134	6.7
60	1996	Weissberg et al.	M	Pleural empyema: 24-year experience [67]	4	Israel	133	4.75
61	2009	Wozniak et al.	M	Treatment of Empyema [68]	2, 4	USA	132	8.8
62	2014	Chung et al.	M	VATS drainage for Empyema [69]	6, 20	South Korea	131	13.1
63	1987	Mandai et al.	J	Treatment of spontaneous bacterial empyema thoracis [70]	4, 16	USA	128	3.46
64	1998	Carey et al.	T	Empyema thoracis: a role for open thoracotomy and decortications [71]	4, 23, 24	UK	128	4.92
65	1991	Poe et al.	B	Pleural Fluid Analysis in Parapneumonic Effusions [72]	1, 4	USA	127	3.85
66	1998	Mandal et al.	M	Primary empyema thoracis [73]	2, 11, 16	USA	126	4.85
67	1999	Grewal et al.	H	VATS in Empyema Management [74]	6, 4	USA	125	5
68	1999	Merry et al.	E	Thoracoscopy in pediatric empyema [75]	5, 17	USA	125	5
69	2012	Marks et al.	AD	Clinical Features of Thoracic Empyema [76]	2, 4	UK	123	10.25
70	2010	Tacconi et al.	G	VATS for empyema thoracis [77]	6	Italy	119	8.5
71	2000	Kercher et al.	B	Thoracoscopic Decortication as First-Line Therapy for Pediatric Parapneumonic Empyema: A Case Series [78]	5, 20, 26	USA	117	4.88
72	2003	Roberts	M	Minimally invasive surgery in empyema [79]	24, 25	USA	116	5.52
73	2003	Satish et al.	T	Management of thoracic empyema in childhood [80]	2, 4, 5, 15, 27	UK	114	5.43
74	2009	Cardillo et al.	G	Chronic postpneumonic pleural empyema [81]	2, 6, 17, 28	Italy	114	7.6
75	1968	Snider et al.	AE	Empyema of the Thorax in Adults: Review of 105 Cases [82]	2, 16, 20	USA	113	2.02
76	1990	Ali et al.	M	Management of empyema thoracis [83]	2, 4, 16	Canada	113	3.32
77	2000	Meier et al.	AF	Rational Treatment of Empyema in Children [84]	4, 5	USA	112	4.67
78	2010	Ahmed et al.	AG	Empyema Thoracis [85]	2, 3	Sudan	112	8
79	1988	Van Way et al.	J	Thoracotomy in empyema treatment [86]	4, 23	USA	111	3.08
80	1995	Stovroff et al.	E	Thoracoscopy in pediatric empyema [87]	5, 18	USA	111	3.83
81	2002	Chen et al.	E	Management of late-stage parapneumonic empyema [88]	1, 4, 5	USA	110	5
82	2003	Hilliard et al.	T	Management of parapneumonic effusion and empyema [89]	1, 4, 5	UK	109	5.19
83	2003	Wells et al.	L	Intrapleural Fibrinolysis for Pediatric Parapneumonic Effusion and Empyema [90]	1, 4, 5,10	USA	107	5.1
84	1987	Foglia et al.	E	Decortication in pediatric empyema [91]	2, 5, 16, 20	USA	106	2.86
85	2002	David et al.	B	Ultrasound-Guided Needle Thoracocentesis vs Chest Tube Drainage in Pediatric Empyema [92]	2, 4, 5, 15	Israel	106	4.82
86	1977	Sherman et al.	P	Management of thoracic empyema [93]	2, 4	USA	104	2.21
87	1945	Burford et al.	AH	Early Pulmonary Decortication in Posttraumatic Empyema [94]	14, 20, 26	USA	100	1.27
88	2009	Palmen et al.	M	Open Window Thoracostomy Treatment of Empyema Is Accelerated by Vacuum-Assisted Closure [95]	2, 4	Netherlands	100	6.67
89	1982	Mayo et al.	M	Acute Empyema in Children [96]	2, 5, 15, 26	USA	99	2.36
90	2006	Kunyoshi et al.	AI	Complicated pneumonias with empyema and/or pneumatocele in children [97]	2, 5,25	Brazil	99	5.5
91	1993	Ferguson	M	Thoracoscopy for empyema, bronchopleural fistula, and chylothorax [98]	2, 3, 17	USA	94	3.03
92	1998	Thourani et al.	M	Empyema treatment: cost-effectiveness analysis [99]	4, 22, 29	USA	94	3.62
93	2004	Kim et al.	P	VATS for postpneumonic pleural empyema [100]	2, 4, 6	South Korea	91	4.55
94	2010	Carter et al.	R	Management of children with empyema [101]	4, 5	USA	90	6.43
95	1963	Yeh et al.	F	Empyema Thoracis: a Review of 110 Cases [102]	2, 3	USA	89	1.46
96	2010	Shahin et al.	O	Management of primary pleural empyema [103]	2, 6, 18, 20	UK	89	6.36
97	1989	Hoff et al.	E	Postpneumonic empyema in childhood [104]	2, 4, 5, 23	USA	88	2.51
98	1981	Mavroudis et al.	J	Improved survival in management of empyema thoracis [105]	2, 4	USA	87	2.02
99	1995	Silen et al.	M	Thoracoscopic debridement of pediatric empyema [106]	2, 4, 5	USA	83	2.86
100	2006	Kalfa et al.	E	Thoracoscopy in pediatric pleural empyema [107]	2, 5, 23	France	82	4.56

**Figure 1:**
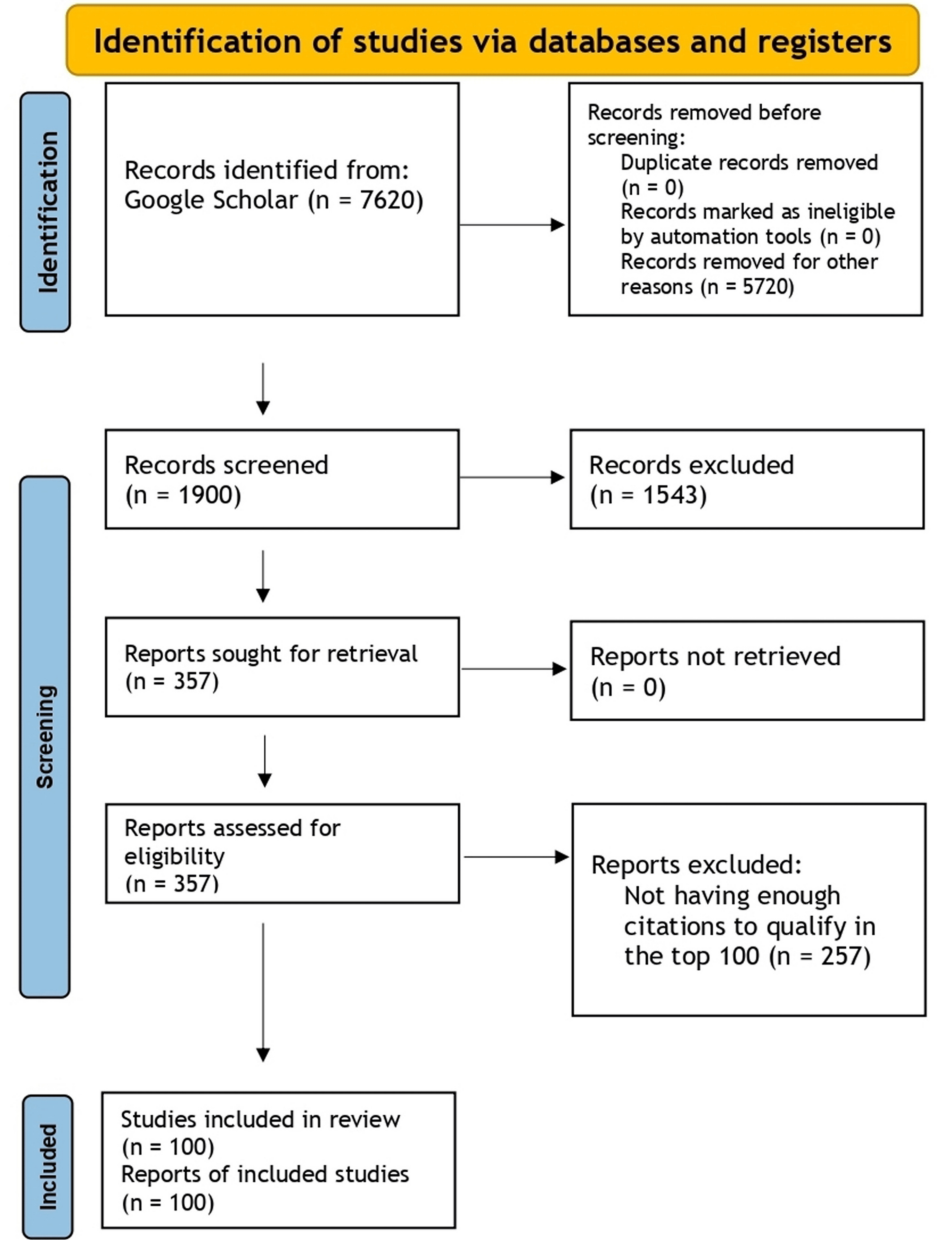
PRISMA 2020 flow diagram illustrating the study selection process for identifying relevant articles on early thoracotomy and decortication in pleural empyema from databases and registries PRISMA – Preferred reporting items for systematic reviews and meta-analyses.

The PRISMA framework guided this bibliometric analysis to ensure transparent and comprehensive reporting of the methodology and findings in the study (Figure 1).

Top 10 Most Cited Articles

The top ten most cited articles collectively amassed 3924 citations, accounting for over 23% of all citations. The publication dates for these articles span from 1993 to 2009, appearing across thirteen distinct journals. Among these, The Annals of Thoracic Surgery featured the highest number of publications. Notably, the most cited work in the review was authored by Light in year 2006, focusing on parapneumonic effusions and empyema (Table 1) [8].

Top 10 Citations per Year (CPY) Metric

The CPY metric represents the ratio of total citations to the article's publication age. This approach mitigates the time bias that allows older articles to accumulate more citations simply due to their age. The aggregate CPY for the 100 analyzed articles is 769.13, with a mean annual citation of 7.69 per article. The minimum and maximum CPY values were 1.27 and 36.11, respectively. The 10 highest-ranking articles were published between 1997 and 2015, indicating a trend towards more recent publications. These top 10 articles received a combined 3509 citations, averaging 350.9 citations each. The CPY for these articles ranged from 15.29 to 36.11, with an average of 19.67 (Table 2).

**Table 2:** Top 10 articles on early thoracotomy and decortication in pleural empyema ranked by citation per year (CPY) metric Sr. No.: serial number, PY: publication year, CC: citation count, CPY: citation per year, USA: United States of America, UK: United Kingdom Journal Title A: Proc Am Thorac Soc, B: CHEST, C: Eur Respir J, D: Am J Respir Crit Care Med, E: J Pediatr Surg, G: Eur J Cardiothorac Surg, I: Clin Infect Dis, N: J Thorac Oncol Category 1: parapneumonic effusions, 2: empyema, 3: review, 4: treatment, 5: pediatric empyema, 6: video-assisted surgery, 9: clinical trial, 10: fibrinolytic therapy, 11: adult, 12: retrospective chart review, 16: infections, 19: oncology, 20: decortication, 21: consensus statement

Sr. No.	PY	Authors	Journal	Article Title	Category	Country	Citation Count (CC)	Citation Per Year (CPY)
1	2006	Light	A	Parapneumonic Effusions and Empyema [8]	1, 2, 3	USA	650	36.11
2	2009	St. Peter et al.	E	VATS versus fibrinolysis for pediatric empyema [14]	5, 6, 9, 10	USA	323	21.53
3	2006	Sonnappa et al.	D	Urokinase and VATS in Childhood Empyema [11]	4, 5, 6	UK	399	21.17
4	2015	Scarci et al.	G	EACTS expert consensus statement for surgical management of pleural empyema [35]	4, 6, 16, 21	UK	182	20.22
5	1997	Wait et al.	B	A Randomized Trial of Empyema Therapy [9]	1, 4	USA	498	18.44
6	2007	Molnar	G	Surgical treatment of adult empyema [16]	3, 11	Hungary	295	17.35
7	1997	Hamm et al.	C	Parapneumonic effusion and empyema [10]	1, 2, 3	Germany	425	15.74
8	2012	Lang-Lazdunski et al.	N	Management of Patients with Malignant Pleural Mesothelioma [33]	4, 19, 20	UK	186	15.5
9	2005	Luh et al.	B	VATS in Complicated Parapneumonic Effusions or Empyemas [17]	1, 6, 12	Taiwan	291	15.32
10	2007	Sahn	I	Diagnosis and Management of Parapneumonic Effusions and Empyema [19]	1, 2, 3	USA	260	15.29

Journals

We included 100 articles from 35 different journals in this review, with The Annals of Thoracic Surgery contributing the most with 22 articles. This journal also led in citation count (CC), accumulating 3125 citations, averaging 142.05 citations per article. CHEST emerged as the journal with the highest average citations per article, with a mean value of 236.69. When considering the CPY metric, The Annals of Thoracic Surgery stood out again with a total of 131.78. The European Journal of Cardio-Thoracic Surgery had the highest mean CPY per article, with their five articles each averaging 12.29 citations annually (Table 3).

**Table 3:** Top journals publishing key research on early thoracotomy and decortication in pleural empyema #: serial number, CC: citation count, CPY: citation per year

#	Journal	Number of Articles	CC	Mean	CPY	Mean
1	The Annals of Thoracic Surgery	22	3125	142.05	131.78	5.99
2	CHEST	13	3077	236.69	115.98	8.92
3	Journal of Pediatric Surgery	11	1567	142.45	70.83	6.44
4	The Journal of Thoracic and Cardiovascular Surgery	6	961	160.17	31.41	5.24
5	European Journal of Cardio-Thoracic Surgery	5	858	171.6	61.46	12.29
6	The American Journal of Surgery	4	506	126.5	15.13	3.78
7	Archives of Disease in Childhood	4	489	122.25	22.11	5.52
8	Pediatrics	3	584	194.67	29.68	9.89
9	American Review of Respiratory Disease	2	408	204	11.75	5.88
10	Clinical Infectious Diseases	2	428	214	21.29	10.65
11	Radiology	2	340	170	11.57	5.79
12	Interdisciplinary CardioVascular and Thoracic Surgery	2	263	131.5	18.79	9.4
13	Pediatric Pulmonology	2	237	118.5	14.17	7.09

Authors

Ninety-three primary investigators authored the top 100 cited articles. The authors with the highest number of publications are listed in Table 4.

**Table 4:** First authors with multiple publications on early thoracotomy and decortication in pleural empyema # : serial number, USA: United States of America, UK: United Kingdom, UCLA: University of California Los Angeles

#	Author	Affiliation	Number of Articles	H-Index	Country
1	Light	Vanderbilt University	2 [8,12]	68	USA
2	Sahn	Medical University of South Carolina	2 [15,19]	74	USA
3	Chen	National Taiwan University Hospital	2 [27,60]	-	Taiwan
4	Hoff	Vanderbilt University	2 [51,104]	-	USA
5	Gates	Children's Hospital and the Ohio State University	2 [55,66]	13	USA
6	Mandal	UCLA School of Medicine	2 [65,73]	-	USA

Categories

**Figure 2:**
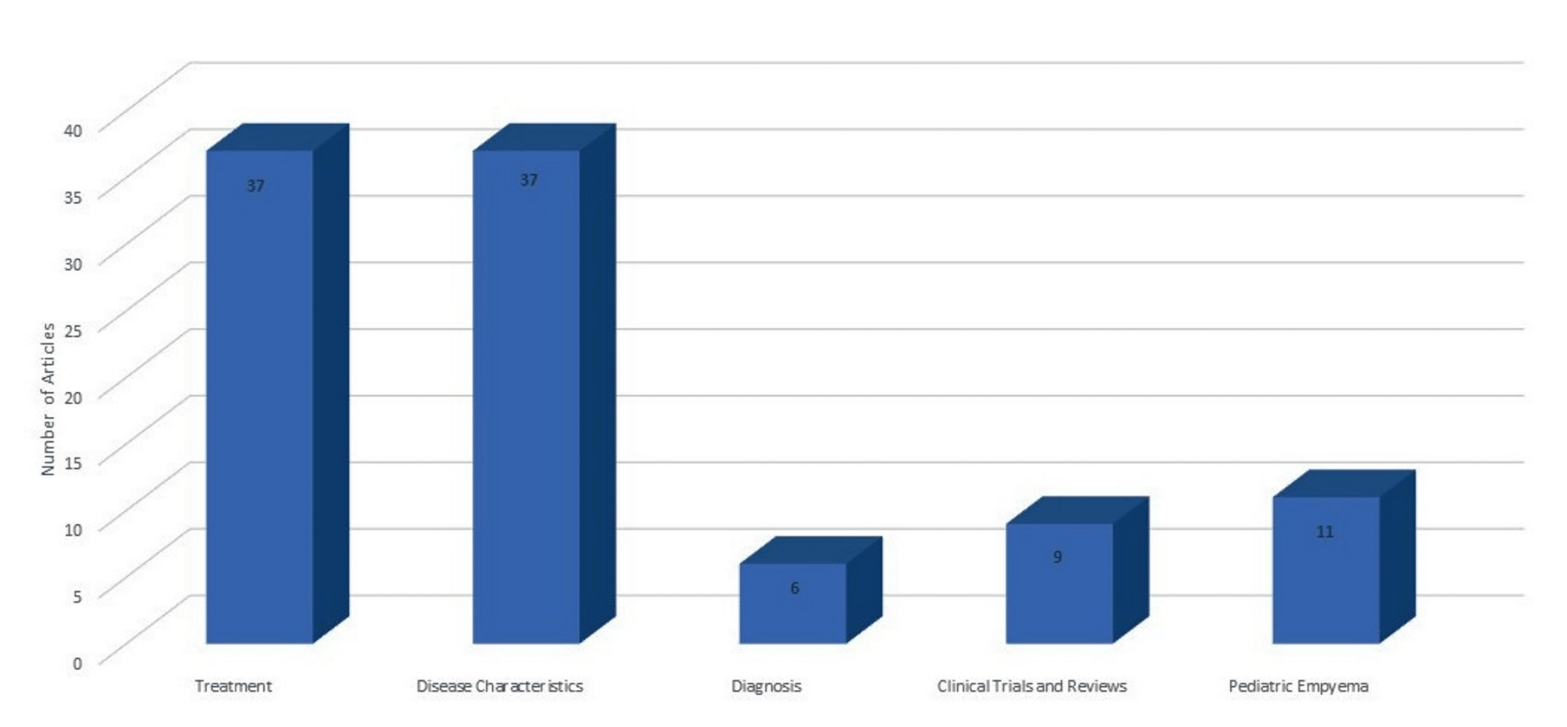
Distribution of research categories in studies on early thoracotomy and decortication for pleural empyema (Image Credits: Dr. Vishal V. Bhende)

The articles were classified based on research focus into treatment (VATS, fibrinolytic therapy, tube thoracostomy drainage, decortication, open thoracotomy, and treatment algorithms), disease characteristics, which encompassed features, causes, and types (para-pneumonic effusions, empyema thoracis, post-traumatic empyema, fibrinopurulent empyema, bacterial empyema, non-tuberculous empyema, post-pneumonic pleural empyema, hemothorax, infections, and acute, chronic, and advanced disease, among others), diagnosis, staging, and disease prognosis (radiological staging, severity scoring, disease classification, imaging, sonographic predictors, and risk, predictive, and prognostic factors), pediatric empyema, and clinical trial and review articles. The most prominent categories widely covered in the literature were those describing the disease characteristics, features, causes, and types and those discussing the various treatment modalities and management strategies, each comprising 37% of all articles. Pediatric empyema was discussed in 11% of the total articles (Figure 2).

Institutions

**Figure 3:**
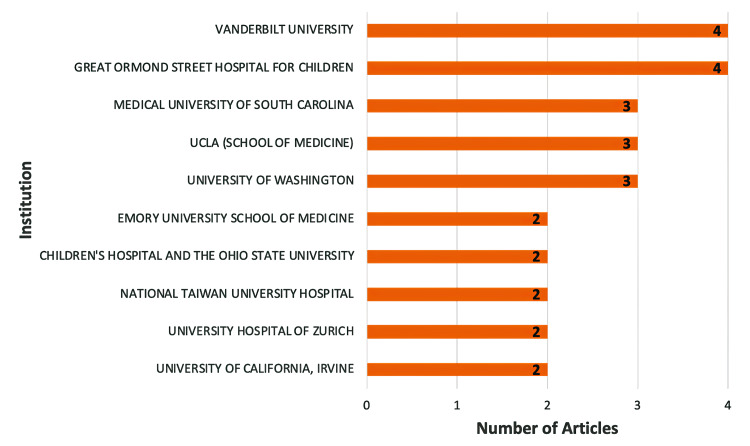
Institutions with the highest contributions to research on early thoracotomy and decortication in pleural empyema UCLA – University of California, Los Angeles (Image Credits: Dr. Vishal V. Bhende)

The top 100 articles on early thoracotomy and decortication in pleural empyema originated from 83 institutions. Of the 83 institutions, 10 had multiple publications in this collection. The most significant contributors to the literature were Vanderbilt University and Great Ormond Street Hospital for Children, with four articles each, and the Medical University of South Carolina, Los Angeles (UCLA) School of Medicine, and the University of Washington, contributing three articles each. The University of California, Irvine, the Children’s Hospital and Ohio State University, the University Hospital of Zurich, National Taiwan University Hospital, and Emory University School of Medicine each contributed two articles (Figure 3).

Countries

**Figure 4:**
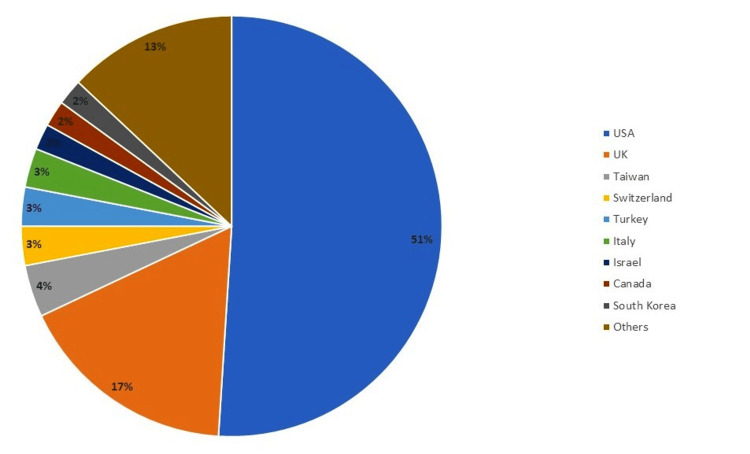
Most prolific countries by contribution on early thoracotomy and decortication in pleural empyema (total 100 articles) (Image Credits: Dr. Vishal V. Bhende)

This review revealed contributions from twenty-two countries, with nine of them having two or more publications. The United States leads significantly, with 52 of the most influential articles on early thoracotomy and decortication in pleural empyema. Following the United States, albeit with a considerable gap, is the United Kingdom, which has published 15 articles. Taiwan had four publications, Switzerland and Turkey had three publications each, whereas Israel, Italy, South Korea, and Canada contributed to two publications each. The honors of one publication were shared by Germany, Hungary, China, Australia, Austria, Greece, South Africa, Sudan, Netherlands Brazil, France, Nepal, and Denmark (Figure 4).

Publication Years

**Figure 5:**
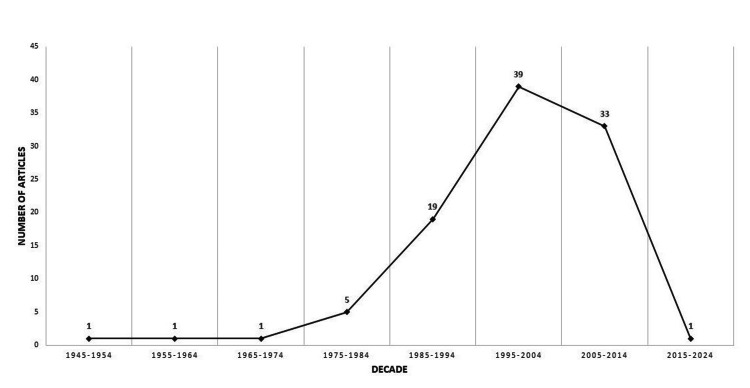
Decadal trend in the publication of articles on early thoracotomy and decortication for pleural empyema (Image Credits: Dr. Vishal V. Bhende)

The top 100 articles spanned a 70-year period from 1945 to 2015. The 1995-2004 decade had the highest publication activity, accounting for 39 articles (39%) of the total, followed by the decade of 2005-2014, which contributed 33 articles (33%) (Figure 5).

Year-to-Year Comparison

**Figure 6:**
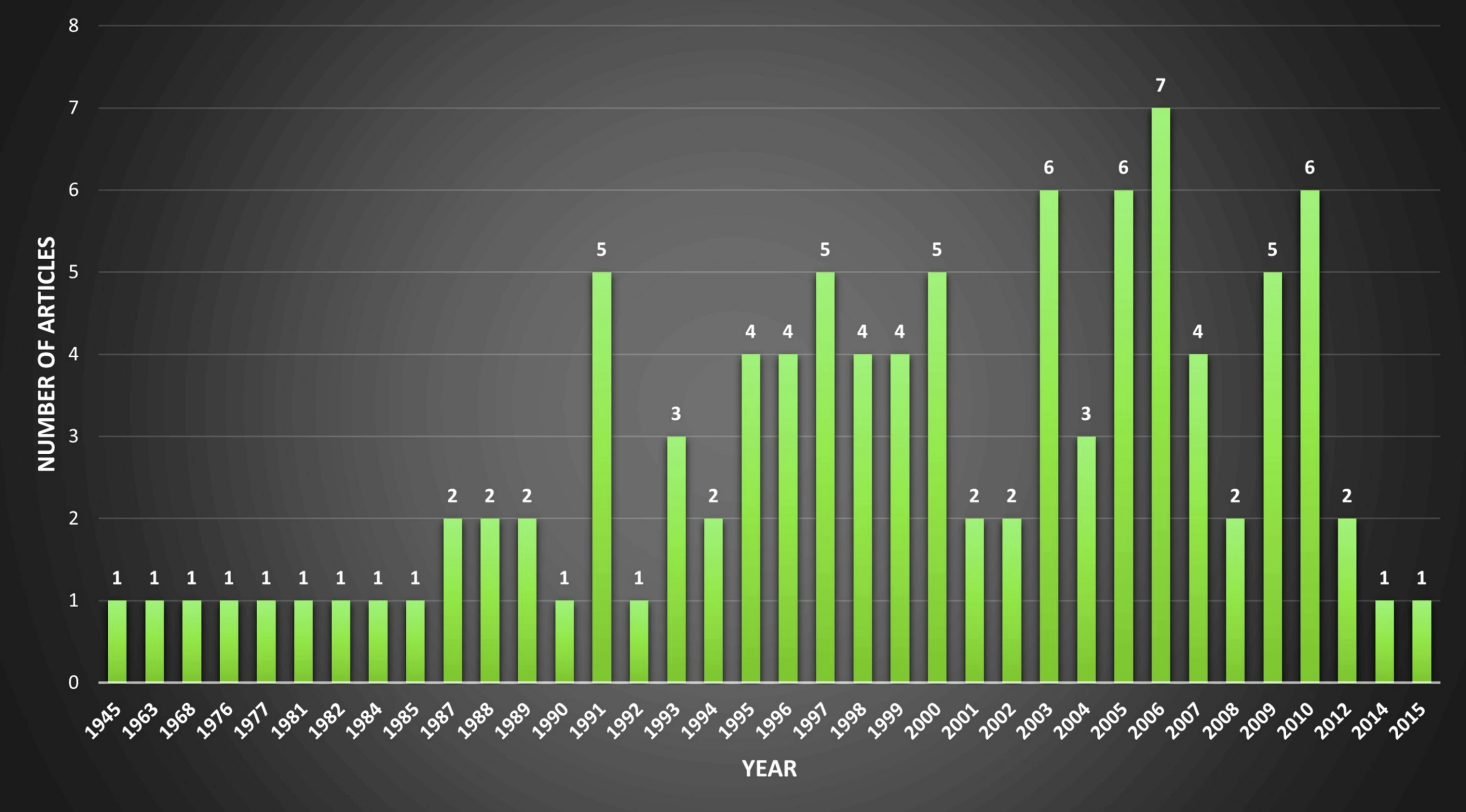
Annual publication rate of articles on early thoracotomy and decortication for pleural empyema (Image Credits: Dr. Vishal V. Bhende)

**Figure 7:**
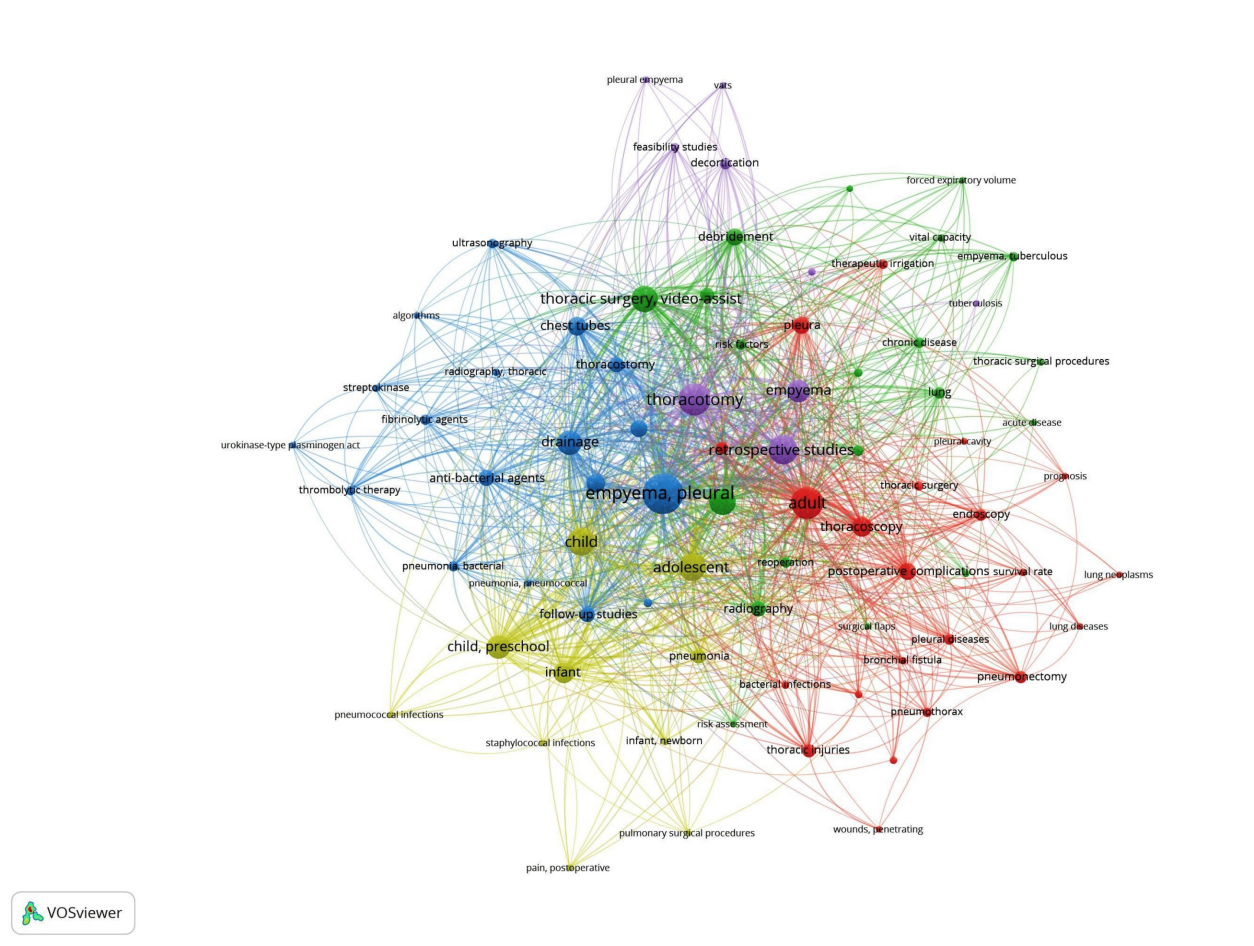
VOSviewer visualization of keyword occurrences and co-occurrences in research on early thoracotomy and decortication for pleural empyema Each node represents a research term and highlights the specific aspect/focus of the research. The node size shows the importance or frequency of each term. Larger nodes represent terms that appear more frequently and/or are more central to the network, and the node colors represent groups/clusters of related terms based on their co-occurrence. Blue cluster: management strategies in empyema; red cluster: adult thoracic conditions, treatment, prognosis, and postoperative complications; green cluster: empyema, assessments, surgical/treatment procedures; yellow cluster: pediatric infections and management; purple cluster: surgical techniques in empyema. The lines between nodes generally represent co-occurrence relationships between terms, and their color typically corresponds to the cluster to which the connecting terms belong. For example, blue lines connect terms in the blue cluster. Therefore, the color of the lines can be interpreted similarly to the color of the clusters. Lines that connect nodes across different clusters have a blended or mixed color appearance. For example, a line connecting a term from the Blue Cluster to a term from the Purple Cluster may show how empyema co-occurs with thoracotomy and retrospective studies. The thickness of the line shows how frequently these terms co-occur or the strength of the relationship between terms based on co-occurrence. Thicker lines indicate a stronger relationship or more frequent co-occurrence between the terms. Thinner lines represent weaker or less frequent connections. (Image Credits: Dr. Vishal V. Bhende)

The year 2006 was the most prolific, with seven articles published, while 2003, 2005, and 2010 contributed six articles each. The years 1991, 1997, 2000, and 2009 were also significantly prolific, as five articles each were published in these years (Figure 6, 7).

Figure 7 shows the keyword occurrences and the relationships between keywords. This figure is important because it gives the viewer certain important information, including areas of research focus and interest and aspects of the research where not much work has been done. The areas with smaller densities represent gaps in the literature and areas of recommendations for future research.

Discussion

Pleural empyema is best managed with early intervention to prevent complications, extensive surgeries, and prolonged hospitalizations [17,108]. However, delayed diagnosis or referral can lead to late treatment, resulting in chronic empyema. For such patients, open thoracotomy and decortication is often recommended. Traditionally, various surgical approaches such as rib resection, Clagett's procedure, open window thoracostomy, thoracoplasty, and ultrasound- or CT-guided percutaneous drainage have been employed [8,99]. Video-thoracoscopy is a new minimally invasive approach for treating chronic empyema [5,26,40,109].

Surgery is often more expensive than simple drainage; however, its higher success rate in empyema patients justifies the expense. St. Peter et al. studied empyema in pediatric patients and found that while VATS decortication was more expensive than fibrinolysis, it did not enhance treatment outcomes. However, their study only evaluated the treatment costs. To accurately assess the costs of treatment, the shortened hospitalization periods and decreased need for subsequent interventions due to treatment failure during surgical decortication should be taken into account [14]. Open thoracotomy and decortication are standard chronic empyema treatments; however, direct comparisons to video thoracoscopy in prospective, randomized studies are lacking. Such research is essential to optimize patient selection for each procedure.

Our bibliometric study of the top 100 most referenced articles on early thoracotomy and decortication in pleural empyema, covering data from 1945 to 2015, revealed key trends and findings. The analysis, based on Google Scholar data, included 16,928 citations from diverse international sources, published by 93 unique first authors across 35 journals. The most cited articles were from The Annals of Thoracic Surgery and CHEST, with the top 10 articles accounting for over 23% of total citations. The most cited article, authored by RW Light of Vanderbilt University in 2006 and published in Clinics in Chest Medicine, has been cited 650 times and discusses parapneumonic effusions and empyema in detail [8].

Contributions came from 22 countries and 83 institutions, with US-based research being dominant, particularly from institutions like Vanderbilt University and the University of California. The literature mainly addressed disease characteristics and treatment modalities, each comprising 37% of the articles, with pediatric empyema featuring in 11% of the articles. The selected articles had a broad citation range (82-650 citations), with an average of 169.28 citations per article. This high citation rate highlights the significant impact of these studies on the field. Our bibliometric analysis has mapped the research landscape on thoracotomy and decortication, identifying gaps and potential areas for future research to enhance diagnostic and treatment protocols.

While the majority of articles focused on disease characteristics and treatment modalities, limited attention was given to the long-term outcomes and quality of life post-surgery. Additionally, the emergence of minimally invasive surgical techniques, such as video-assisted thoracoscopic surgery (VATS), in recent years calls for updated analyses that evaluate the comparative effectiveness of these modern approaches versus traditional thoracotomy. Future research should also explore the role of interdisciplinary care, integrating pulmonology, surgery, and critical care perspectives to develop holistic management strategies for pleural empyema.

Limitations

The present study has some notable limitations. First, high citation counts may not necessarily indicate significant impact, as articles may be cited for critical analysis or to highlight weaknesses. Additionally, CC can be skewed as recent studies may not have as much time as the older ones to garner citations. To address this limitation, we utilized the CPY metric in our review.

Second, this review was conducted using only the Google Scholar database. Although Google Scholar covers more sources than individual databases like PubMed, Scopus, and Web of Science, it has limitations. Web of Science, for instance, contains the oldest publications, dating back to 1900, [110] whereas Google Scholar captures articles from 1945 onwards. Consequently, older studies available in Web of Science but not in Google Scholar were excluded from this analysis.

## Conclusions

This bibliometric analysis of articles on early thoracotomy and decortication in pleural empyema offers valuable insights into the current research landscape. It emphasizes the importance of ongoing high-quality studies to enhance our understanding, diagnostic approaches, and treatments for this condition. The analysis identifies key articles, influential authors, leading institutions, contributing countries, and prominent journals, noting a predominance of research on treatment and disease characteristics, with limited focus on radiology. Future research should address these gaps by incorporating radiological techniques to improve diagnostic accuracy and therapeutic decision-making. Collaborative efforts are crucial to developing innovative strategies for better patient outcomes.
